# Exploring the Interplay Between Kidney Dysfunction and Cardiovascular Disease

**DOI:** 10.3390/medsci13020080

**Published:** 2025-06-18

**Authors:** Rajesh Yadav, Aqsa Kaim Abubakar, Richa Mishra, Saurabh Gupta, Neelesh Kumar Maurya, Vivek Kumar Kashyap, Sarvesh Rustagi, Deependra Pratap Singh, Sanjay Kumar

**Affiliations:** 1 Department of Dialysis Technology, Sharda School of Allied Health Sciences, Sharda University, Greater Noida 201310, Uttar Pradesh, India; 2 Department of Computer Engineering, Parul Institute of Engineering and Technology (PIET), Parul University, Ta. Waghodia, Vadodara 391760, Gujarat, India; 3 Department of Biotechnology, GLA University, Mathura 281406, Uttar Pradesh, India; 4 Department of Nutrition and Dietetics, Sharda School of Allied Health Sciences, Sharda University, Greater Noida 201310, Uttar Pradesh, India; 5 Medicine and Oncology Integrated Service Unit, Division of Cancer Immunology and Microbiology, School of Medicine, University of Texas Rio Grande Valley, McAllen, TX 78504, USA; 6 South Texas Center of Excellence in Cancer Research (ST-CECR), University of Texas Rio Grande Valley, McAllen, TX 78504, USA; 7 Department of Food Technology, School of Applied and Life Science, Uttaranchal University, Dehradun 248007, Uttarakhand, India; 8 Department of Biotechnology, Graphic Era (Deemed to be University), Dehradun 248002, Uttarakhand, India; 9 Department of Life Sciences, Sharda School of Basic Science and Research, Sharda University, Greater Noida 201310, Uttar Pradesh, India

**Keywords:** cardiovascular disease, chronic kidney disease, nitric oxide signaling, hemodialysis

## Abstract

This article reveals the various types of complications that are associated with dialysis and kidney-associated disease, including left ventricular hypertrophy, heart failure, vascular heart disease, arrhythmias, diabetes mellitus, intradialytic hypertension, and coronary heart disease. The molecular mechanisms underlying the development of cardiovascular disease in patients with chronic kidney disease (CKD), including the role of nitric oxide (NO) signaling, have been extensively studied. Patients suffering from CKD need treatment with hemodialysis at the end stages. The kidney is considered the chief excretory organ in humans, which excretes various types of waste materials from the body and balances the acid–base ratio, due to which its role in homeostasis has been considered. When kidneys fail to function properly due to various diseases, hemodialysis plays the role of the kidneys. This procedure involves removing a patient’s blood, filtering it through a dialyzer to remove waste products, and returning the cleaned blood to the body. However, for the hemodialysis procedure, fistula formation is necessary, which is created by specific surgery in which the radial artery and superficial vein are connected in the forearm, near the wrist or elbow. This arteriovenous (AV) fistula creation fails sometimes and causes complications. The prolonged use of hemodialysis procedures and improper care also lead to many complications in chronic kidney patients, which have been discussed in detail in this review article.

## 1. Introduction

Cardiovascular disease (CVD) is a significant global health concern affecting millions of people worldwide. CVD is also a major cause of death worldwide. According to the American Health Association 2023 update, globally, CVD accounted for an estimated 0.92 million deaths in 2020 [1,2]. In 2019, it was responsible for an estimated 18.6 million deaths, representing 32% of all global deaths [3]. While mortality rates have declined in some high-income countries due to improved risk factor management and treatment, CVD remains a major health challenge in many low and middle-income countries. The cardiovascular disease burden in low and middle-income countries is escalating, imposing significant economic and health costs [4]. There is an imperative need to prioritize implementing existing cost-effective policies and interventions to mitigate this growing challenge.

Chronic kidney disease (CKD) patients face a substantially elevated risk of heart disease and stroke. Early-stage CKD patients face a significantly increased risk of heart attacks and strokes. This risk is further amplified in advanced-stage CKD (stages 4–5), where CVD is the primary cause of death, surpassing end-stage renal disease (ESRD) [5]. Approximately half of patients with advanced-stage chronic kidney disease (stages 4–5) exhibit cardiovascular disease. Moreover, cardiovascular mortality constitutes approximately 40–50% of all deaths in this patient population, significantly exceeding the 26% observed in people having normal kidney function.

Improving Global Outcomes (KDIGO) is a global organization focused on developing and implementing evidence-based clinical practice guidelines in kidney disease [6]. According to KDIGO, CKD is defined as the presence of kidney damage or a glomerular filtration rate (GFR) of less than 60 mL/min/1.73 m^2^ for three months or longer, regardless of the underlying cause [7,8]. According to the World Health Organization (WHO) and Pan American Health Association (PAHA), chronic kidney disease, also known as chronic kidney failure (CKF), is a progressive condition that involves a gradual loss of kidney function [9]. The kidneys are vital organs for filtering waste products and excess fluid from the blood, which are subsequently excreted in the urine. As CKD progresses to an advanced stage, the kidneys become increasingly impaired, leading to the accumulation of dangerous levels of fluid, electrolytes, and waste products in the body [10]. Kidney damage in various kidney diseases can be assessed by albuminuria, defined as an albumin-to-creatinine ratio exceeding 30 mg/g in two out of three spot urine specimens. As the kidneys become less efficient, waste products and excess fluid build up in the body, leading to various health complications [11]. Dialysis is a treatment that replaces the function of failing kidneys. It involves blood filtering to remove waste products and excess fluid [12]. Dialysis is a crucial medical intervention that allows individuals with kidney failure to survive and maintain a reasonable quality of life. Elderly people worldwide face the problem of chronic kidney disease, which is the most widespread disease globally. Kidney damage is one of the crucial reasons for CKD. The glomerular filtration rate serves as the principal determinant in staging chronic kidney disease, which is classified into five distinct stages [7]: Stage 1, early kidney damage with normal or increased kidney function (GFR ≥ 90 mL/min/1.73 m^2^); stage 2, mild kidney damage with slightly reduced kidney function (GFR, 60–89 mL/min/1.73 m^2^); stage 3, moderate kidney damage with significantly reduced kidney function (GFR, 30–59 mL/min/1.73 m^2^); stage 4, severe kidney damage with greatly reduced kidney function (GFR, 15–29 mL/min/1.73 m^2^); stage 5, kidney failure with minimal to no kidney function (GFR < 15 mL/min/1.73 m^2^), frequently called an end-stage renal disease (ESRD) [13]. Dialysis is performed in chronic kidney disease (CKD) when the GFR < 15 mL/min/1.73 m [14]. In CKD, toxins and water are stored due to compromised kidney function, and dialysis is favored to treat ESRD and remove stored toxins from the body.

**Figure 1:**
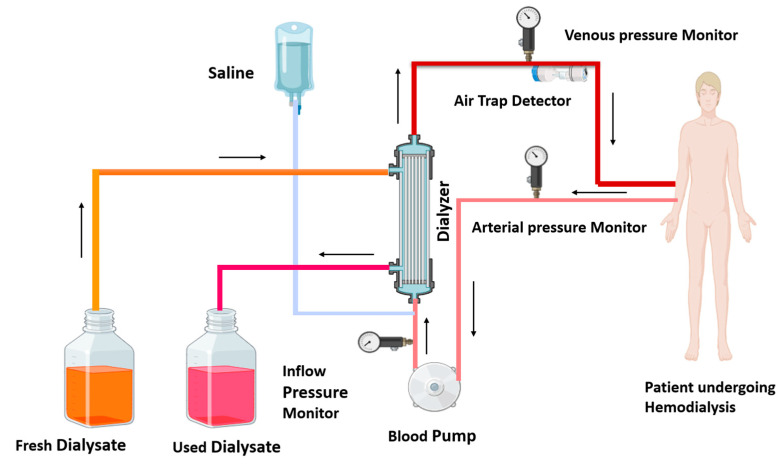
Diagram of hemodialysis procedure: in the hemodialysis procedure, impure blood is removed from the patient, passes through the blood pump, and enters the dialyzer; waste toxins of the blood are removed from the dialyzer, and the purified blood recirculates in the body. The used dialysate is collected in a container, and fresh dialysate is supplied into the dialyzer, as shown by the arrows. Arterial and venous pressure monitors detect pressure in arterial and venous blood. The air trap monitor detects the air bubble in recirculating blood. A saline solution enters the dialyzer with impure blood and cleanses the blood.

Government health insurance programs in Australia, Canada, the United Kingdom, and the United States cover the cost of dialysis for eligible individuals. Dialysis is a powerful tool for molecular separation in labs. Dialysis is of two kinds: one is hemodialysis, and the other is peritoneal dialysis [12]. In India and middle-income countries, hemodialysis achieves more success than peritoneal dialysis [15,16]. Hemodialysis employs a semi-permeable membrane to facilitate the diffusion of solutes and the ultrafiltration of excess fluid from the blood. Toxin and waste material diffuse from a high concentration (blood) to low concentration (dialysate). In dialysis, ultrafiltration is achieved by increasing the hydrostatic pressure on one side of the dialyzer membrane [12]. Ultrafiltration is facilitated by applying negative pressure to the dialysate compartment, which induces the movement of water and dissolved solutes from the blood into the dialysate [17]. This process removes several liters of excess fluid during a typical 4 h dialysis session. In 2010, roughly 2.5 million individuals worldwide required chronic renal replacement therapy [12]. This treatment is indispensable for managing end-stage renal disease, a global health concern predominantly caused by diabetes mellitus and hypertension. Patients with kidney disease undergo dialysis and experience various types of complications, which have been discussed in detail. Figure 1 illustrates the ray diagram of the hemodialysis procedure.

## 2. Molecular Mechanism of Development of Cardiovascular Disease in CKD Patients

Cardiovascular disease can develop in CKD patients by (a) vascular calcification, (b) inflammation and oxidative stress, (c) alteration of ion channels or (d) dysregulation of nitric oxide signaling. In addition, obesity hyperlipidemia and cigarette smoking can enhance the risk of CVD in CKD patients.

(a) Vascular Calcification: The mechanisms underlying vascular calcification in CKD include increased phosphate levels, abnormal calcium metabolism, and the role of inflammation. These contribute to arterial stiffness and increased cardiovascular risk in CKD patients [18].
(b) Inflammation and Oxidative Stress: Chronic inflammation in CKD promotes oxidative stress, which damages endothelial cells and contributes to atherosclerosis. Cytokines and other inflammatory mediators play a significant role in this process, facilitating cardiovascular deterioration. More production of reactive oxygen species (ROS), and reduction in antioxidants in dialysis procedures also increase oxidative stress [19].
(c) Alterations in Ion Channels: Changes in cardiac ion channels and repolarization processes can lead to arrhythmias in CKD patients. These alterations are influenced by electrolyte imbalances and the effects of uremic toxin. The uremic toxin also increases calcification in blood vessels and is responsible for inflammation [20].
(d) Nitric Oxide Signaling: The role of nitric oxide (NO) in regulating vascular tone and its dysregulation in CKD leads to endothelial dysfunction, contributing to hypertension and cardiovascular complications. Impaired NO availability can result from oxidative stress and altered metabolic pathways in CKD patients. NO signaling and dysregulation leads to the development of CVD, which is described below in detail.

### 2.1. Role of Nitric Oxide in the Development of Cardiovascular Disease

**Figure 2:**
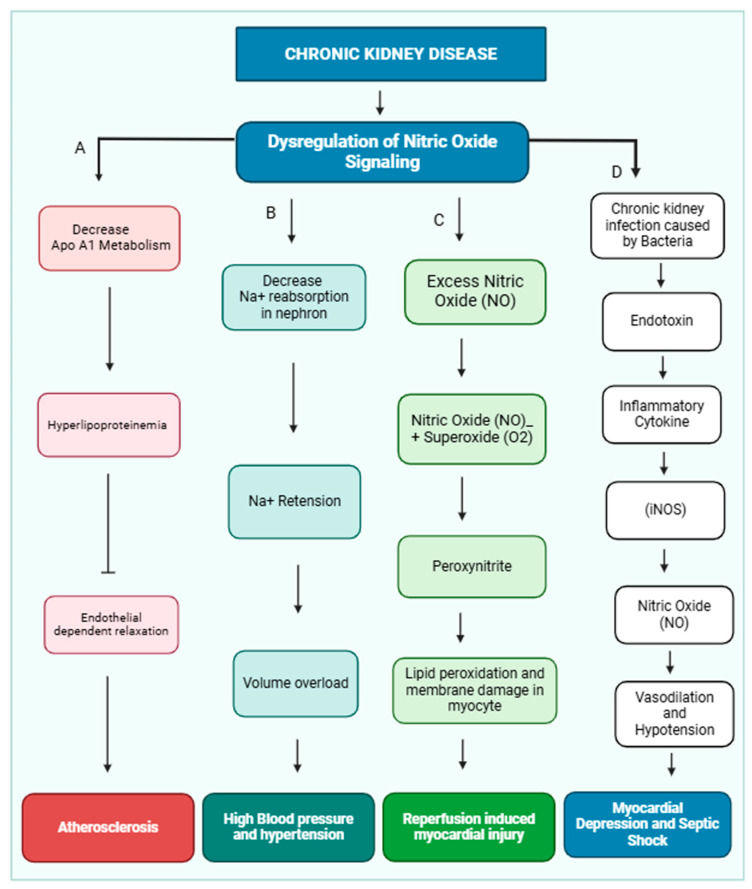
Dysregulation of nitric oxide signaling pathway and development of cardiovascular disease in CKD patients. In CKD, the kidney is not functioning properly due to dysregulation of nitric oxide signaling. (A) Dysregulation of nitric oxide inhibits ApoA1 lipoprotein metabolism, and accumulation of HDL causes hyperlipidemia, which inhibits endothelial-dependent relaxation of the blood vessel and causes atherosclerosis. (B) Dysregulation of nitric oxide inhibits sodium ion reabsorption in different parts of the nephron, which causes sodium ion retention and creates volume overload and hypertension. (C) Excess nitric oxide combined with superoxide (O2^−^) forms peroxynitrite, which causes damage to the myocyte membrane and causes reperfusion-induced myocardial injury. (D) Chronic kidney infection can lead to an accumulation of endotoxins. These endotoxins trigger the release of inflammatory molecules, which in turn stimulate the production of nitric oxide. Nitric oxide can vasodilate and reduce blood pressure(hypotension). This can result in reduced heart function and cause myocardial depression and septic shock.

Nitric oxide (NO) is crucial in maintaining cardiovascular health by regulating vascular tone, myocardial contractility, and endothelial integrity [21]. Additionally, it inhibits platelet aggregation. Dysregulation of NO production has been implicated in the development of several cardiovascular diseases, including essential hypertension, reperfusion injury, atherosclerosis, and myocardial depression associated with septic shock [22]. NO release from the endothelial layer also maintains blood pressure. Various physiological factors, including platelet-derived substances like ADP and serotonin, thrombin, and shear stress, activate NO release. Shear stress is a primary stimulus for releasing NO, and this effect may be mediated by a transient receptor potential vanilloid type IV (TRPV4) channel, which is located in the nephron and maintains osmolarity [23]. TRPV4 channels are nonselective cationic channels and preferably allow calcium ions inside the cell, and shear stress is an exogenous activator of the TRPV4 channel [24,25]. NO exerts a more substantial influence on the function of thicker arteries, which experience significant fluctuations in pulsatile blood flow and shear stress [26,27]. NO signaling leads to hypertension and other cardiac diseases, which are shown below (Figure 2).

### 2.2. Kidney Dysfunction and Atherosclerosis in Coronary Artery

High levels of low-density lipoprotein (LDL) cholesterol and triglycerides, along with reduced levels of high-density lipoprotein (HDL) cholesterol, are significant risk factors for atherosclerotic disease [28]. Emerging evidence suggests that these lipid abnormalities may also contribute to the development of CKD [29]. Apolipoprotein A1 and B1 are associated with CKD [30]. Apolipoprotein A1 is the cornerstone protein of HDL, while apolipoprotein B is a key component of IDL, VLDL, and LDL particles. Studies indicate that elevated levels of lipoproteins can impair endothelial function, even in the absence of atherosclerosis [31]. Endothelial dysfunction and hypercholesterolemia may promote atherogenesis. Takahashi et al. demonstrated in vitro that HDL, LDL, and VLDL can inhibit endothelium-dependent relaxation [32]. Impaired endothelium-dependent relaxation in coronary arteries causes atherosclerosis [33].

### 2.3. Kidney Dysfunction and Septic Shock

Excessive NO production may contribute to the hypotension and myocardial depression characteristic of septic shock [34]. Severe Gram-negative bacterial infections in the kidney lead to the release of endotoxin, which is a lipopolysaccharide and is present in bacterial cell walls [35]. It is made up of three parts, the outer core polysaccharide (O-antigen), inner core oligosaccharide, and Lipid A. Endotoxins promote the release of cytokines, which are pro-inflammatory and activate inducible nitric oxide synthase (iNOS) in macrophages and vascular smooth muscle cells [36]. Various cytokines have been shown to induce iNOS gene expression [37]. Endotoxins can also stimulate iNOS gene expression in both blood vessel muscles and macrophage cells. iNOS synthesizes an abundance of NO, resulting in vasodilation and hypotension associated with septic shock.

### 2.4. Kidney Dysfunction and Reperfusion Injury

Reperfusion injury, a condition where tissue damage occurs upon restoration of blood flow after a period of ischemia, can affect various organs, particularly the heart [38]. This injury can lead to serious consequences such as myocardial infarction and stroke [39]. The mechanism of reperfusion injury involves the release of superoxide from activated leukocytes. Superoxide anion (O2^−^) is believed to play a significant role in this process, as evidenced by the protective effect of superoxide dismutase (SOD). During reperfusion, both O2^−^ and NO are produced, which can combine and produce peroxynitrite, a highly reactive oxidant species causing lipid peroxidation and protein damage [40].

### 2.5. Effect of Nitric Oxide on Tubular Reabsorption in Chronic Kidney Disease (CKD)

The kidney, a vital organ, plays a pivotal role in maintaining the body’s internal balance, a state known as homeostasis. NO is widely recognized for inhibiting Na^+^ reabsorption in different parts of the nephron [41]. However, the specific impact of NO can vary depending on various factors, including the acute or chronic nature of the condition, the experimental setting (in vivo, ex vivo, or in vitro), and the species under study. Additionally, the influence of NO on sodium handling appears to be modulated by hormonal factors, particularly through interactions with the renin–angiotensin–aldosterone system [42]. In the different segments of the nephron, sodium and other minerals are transported [43]. In the proximal convoluted tubule (PCT), (tubular segment of nephron), a sodium hydrogen exchanger (NHE3) is located in the apical membrane of tubular cells, which allows sodium ions inside the tubular cells from the lumen by the exchange of hydrogen ions (H^+^). In PCT, sodium and glucose cotransporters (SGLTs) are also located, which allows sodium and glucose to be simultaneously stored inside the tubular cell for reabsorption [38]. In CKD patients, NO produced from neuronal nitric oxide synthase (nNOS) and endothelial nitric oxide synthase (eNOS) inhibit NHE3, Na-K pump/Na-K-ATPase, and Na^+^/HCO3^−^ cotransporters found in the basolateral surface of tubular cells [44]. In this way, sodium reabsorption from the PCT of the nephron is prevented.

**Figure 3:**
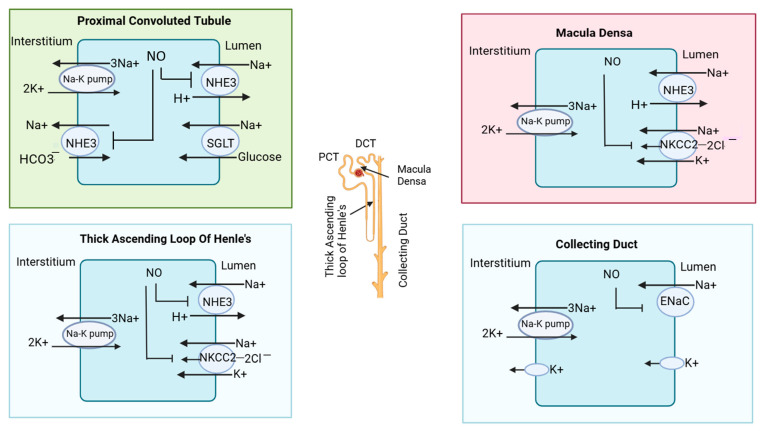
Effect of nitric oxide on transporters in nephrons. In the proximal convoluted tubule, nitric oxide (NO) inhibits sodium reabsorption by blocking the sodium hydrogen exchanger (NHE3). In macula densa, NO inhibits the sodium hydrogen exchanger and (Na^+^-K^+^-2Cl^−^) cotransporter (NKCC2). In the thick ascending loop of Henle, NO also blocks the NKCC2 cotransporter; in the collecting duct, it blocks the epithelial sodium channel (ENaC). The different segments of the nephron are also illustrated in the structure of the nephron in the central region.

In the thick ascending loop of Henle’s basically minerals (Na^+^, K^+^, 2Cl^−^), absorption occurs via NKCC2 transporters [45]. In CKD patients, eNOS-derived NO inhibits NHE3 and may also influence the apical Na^+^-K^+^-2Cl^−^ cotransporter (NKCC2). Furthermore, eNOS-derived NO inhibits NKCC2 in macula densa cells [46,47]. The macula densa is a specialized group of cells located at the junction of a thick ascending loop and early distal convoluted tubules. While nNOS expression has been observed in the early distal tubule, the specific effects of NO on transporters like the Na^+^/Cl^−^ cotransporter in this segment remain less understood. Lastly, in collecting duct cells, nNOS-derived NO can inhibit the epithelial sodium channel (ENaC) [42]. The nitric oxide-mediated inhibition of ion transporters in different parts of the nephron is illustrated in Figure 3.

### 2.6. Molecular Mechanism of Cardiorenal Syndrome

The American Heart Association has issued the following scientific statement about cardiorenal syndrome: “Cardiorenal syndrome (CRS) describes the interplay between heart and kidney function, where acute or chronic problems in one can led to acute or chronic problems in the other [48]”. The development and progression of cardiorenal syndrome (CRS) are driven by a complex interplay of hemodynamic (reduced cardiac output, volume overload), neurohumoral (renin–angiotensin–aldosterone system, sympathetic nervous system), inflammatory, and oxidative mechanisms [49]. Reduced cardiac output, decreased arterial pressure, and increased central venous pressure due to systemic venous congestion and impaired renal perfusion trigger a cascade of neurohumoral responses, including activation of the renin–angiotensin–aldosterone system (RAAS), the sympathetic nervous system (SNS), and the release of antidiuretic hormone (arginine vasopressin). Additionally, RAAS upregulation contributes to a pro-inflammatory state and increased oxidative stress, further exacerbating organ damage [19]. These compensatory neurohumoral responses, designed to maintain perfusion to vital organs in a compromised circulatory system, can inadvertently initiate a self-sustaining cycle of fluid retention, venous congestion, tissue hypoperfusion, inflammation, and oxidative stress [50]. This cycle can further deteriorate both cardiac and renal function. Cardiorenal syndrome can be classified into different types based on primary organ dysfunction and the temporal relationship between cardiac and renal events: type 1—acute kidney injury (AKI) secondary to acute heart failure; type 2—chronic kidney disease (CKD) leading to heart failure; type 3—acute or chronic heart failure leading to AKI; type 4—chronic heart failure and CKD, each contributing to the worsening of the other; type 5—acute decompensation of chronic heart failure and worsening renal function [49].

## 3. Chronic Complications Associated with CKD and Dialysis

### 3.1. Intradialytic Hypertension

Hypertension is one of the classical risk factors among chronic kidney disease patients and hemodialysis patients [51]. It refers to a significant rise in blood pressure that occurs during a hemodialysis session, typically defined as an increase of 10–20 mm (about 0.79 in) Hg systolic or an absolute value above 140/90 mm (about 3.54 in) Hg (Agarwal, 2010). It is a common complication among hemodialysis patients, affecting approximately 15–20% of cases [52].

The exact mechanisms underlying intradialytic hypertension are not fully understood, but several factors likely contribute, including volume overload, sympathetic nervous system activation, endothelial dysfunction, and electrolyte imbalances [53]. Hypertension may also be associated with intestinal microbiota. The study published in the journal *Kidney International Reports* in 2020 indicated the role of specific gut microbiota in the development of intradialytic hypertension. It reveals that alterations in the gut microbiome composition were associated with intradialytic hypertension in hemodialysis patients [54]. The researchers observed differences in the abundance of certain bacterial species between patients with intradialytic hypertension and those without, suggesting a potential link between gut microbiota dysbiosis and blood pressure dysregulation during hemodialysis [54]. Reduced nephron function stimulates the renin–angiotensin–aldosterone system, resulting in sodium retention and fluid volume expansion. These changes contribute to hypertension by triggering various mechanisms that elevate blood pressure and activate the inflammatory immune system. In CKD patients, sodium can accumulate in muscles and skin without causing proportional water retention. This non-osmotic accumulation is related to the severity of kidney dysfunction.

Elevated levels of steroid compounds in CKD patients can contribute to hypertension by impairing the vasodilatory mechanism [55]. Vascular stiffness, a result of inflammation and calcification due to hyperphosphatemia and secondary hyperparathyroidism, is a significant factor in elevated systolic blood pressure in CKD patients [56,57]. Disruption of nitric oxide (NO)-mediated blood vessel homeostasis occurs when the enzyme nitric oxide synthase is inhibited. This inhibition is caused by the accumulation of endogenous inhibitors, such as asymmetric dimethylarginine (ADMA). The combined effect of these accumulated inhibitors and increased endothelin levels contributes to hypertension and the development of atherosclerosis [58].

### 3.2. Left Ventricular Hypertrophy (LVH)

Hemodialysis patients with chronic kidney disease have a higher amount of fibroblast growth factor 23 (FGF23), which causes left ventricular hypertrophy (LVH) [57]. The results of a previous study in 2009 on 124 patients with end-stage renal disease also correlate with the results from the Nilsen study [59,60]. Phosphate and mineral homeostasis are controlled by FGF23 produced by osteocyte cells and for this reason, they are called phosphotropic hormones. FGF23 is not only associated with LVH but also associated with atrial fibrillation and heart failure conditions. LVH is characterized by the thickening (hypertrophy) of the myocardium (muscle) of the heart’s left ventricle. It typically occurs as a response to chronic pressure overload, such as hypertension or other conditions that increase the workload on the left ventricle [61]. LVH is considered a significant risk factor for heart-associated diseases, including arrhythmias and heart failure [62].

FGF23 is a protein made by bone cells that helps control the phosphorus level in our body. It works with other hormones like the parathyroid hormone to regulate phosphorus levels [63]. The parathyroid hormone also reduces phosphate reabsorption from the kidney (proximal convoluted tubule) and intestine, while vitamin D helps to absorb calcium and phosphate.

**Figure 4:**
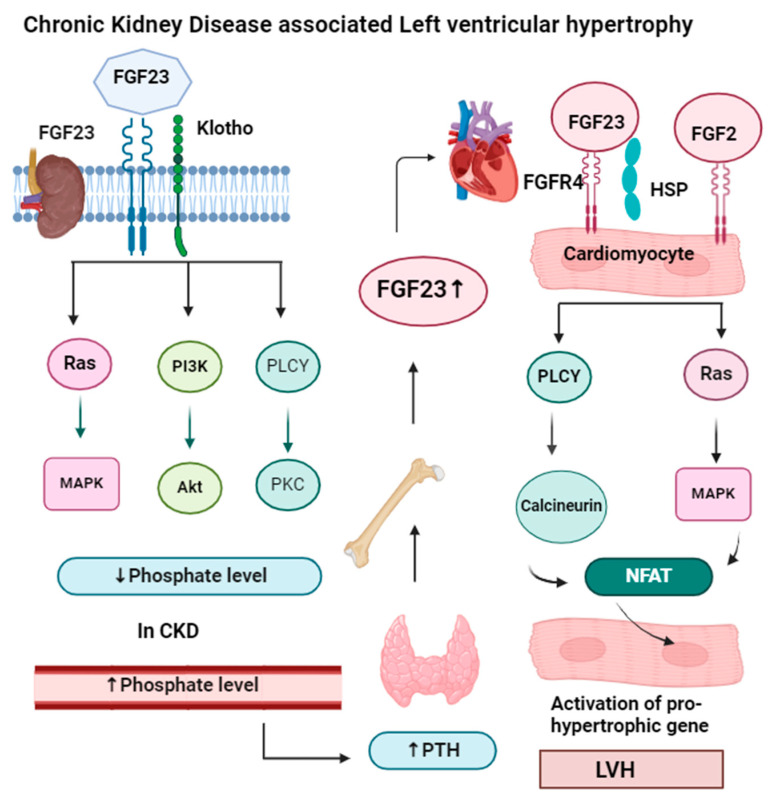
In the kidney and parathyroid glands, FGF23 signaling requires the presence of FGFR (fibroblast growth factor receptor) and the coreceptor klotho. The binding of the FGF23-klotho complex to FGFR triggers auto-phosphorylation of the receptor tyrosine kinase. This activation initiates signaling through three major pathways: Ras-MAPK, PI3K-Akt, and PLCγ-PKC. These pathways help the kidneys maintain a phosphate balance. In chronic kidney disease, when phosphate levels increase in the blood, it activates the parathyroid gland to secrete parathyroid hormone, which activates bone to release more FGF23, which binds in cardiomyocytes along with the HSP receptor. In cardiomyocytes, FGF23 signaling requires FGFR (fibroblast growth factor receptor) and heparan sulfate proteoglycans (HSP) as a coreceptor. FGF23 binding to FGFR4 on cardiomyocytes stimulates autophosphorylation of the receptor tyrosine kinase independently of klotho. FGF23 increases NFAT, which enters the nucleus of the cardiomyocyte and increases the expression of hypertrophic genes, result left ventricular hypertrophy. Apart from FGF23, FGF2 also causes hypertrophy by the Ras-MAP signaling pathway.

FGF23 needs another protein called alpha-klotho for phosphate homeostasis. FGF23 lowers phosphate levels by preventing phosphate reabsorption from the proximal convoluted tubule (PCT) of the nephron and reducing the production of active vitamin D (1,25-dihydroxyvitamin D). In CKD, both FGF23 and parathyroid levels increase, so they act as markers of CKD, but FGF23 levels rise before other markers like parathyroid hormone and phosphorus in people with early kidney disease [64]. High levels of FGF23 have been linked to worse kidney disease, heart problems, and an even higher risk of death. FGF23 signaling in the kidney and parathyroid glands requires FGFR and the coreceptor klotho. FGF23 binds to FGFR and maintains phosphorus balance by upregulating gene expression of the parathyroid hormone, and downregulating gene expression of vitamin D. In the heart muscle, FGF23 signaling also involves FGFR, which requires heparan sulfate proteoglycan (HSP) coreceptors instead of klotho. But in heart muscle, the PLCγ-calcineurin pathway is dominant, and FGF23 increases the nuclear factor of activated T cells (NFAT) and enhances left ventricular hypertrophy. The mechanism of left ventricular hypertrophy by FGF23 is illustrated in Figure 4.

### 3.3. Cardioprotective Role of Klotho

**Figure 5:**
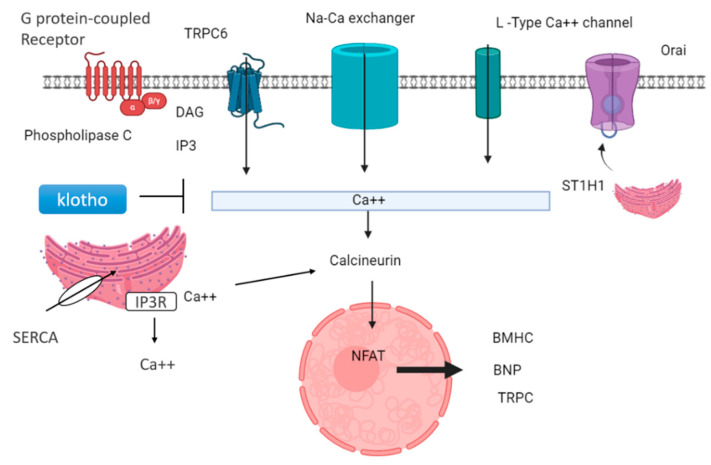
Cardioprotective role of klotho and downregulation of genes related to hypertrophy: GPCR-mediated signaling can activate PLCβ and PLCγ, leading to diacylglycerol (DAG) and inositol 3-phosphate (IP3). This triggers the release of calcium ions from intracellular stores. Various calcium channels, including L-type calcium channels, Orai, sodium–calcium exchangers, and TRPC6, facilitate calcium influx into the cell. STIM1, a calcium sensor, is crucial in store-operated calcium entry (SOCE) through TRPC channels. Additionally, the sarcoplasmic reticulum releases calcium ions, activating calcineurin, which stimulates the transcription factor NFAT, regulating the expression of genes involved in hypertrophy, such as β-myosin heavy chain (β-MHC) and brain natriuretic protein (BNP). Klotho, a protein with anti-aging properties, inhibits TRPC6 channel calcium ion release and subsequently suppresses calcineurin activity, leading to downregulation of hypertrophy-related genes.

Lower levels of klotho protein are found in people with kidney disease; therefore, they act as a marker in CKD. Decreased klotho levels and elevated FGF23 levels are signs of poor outcomes for people with kidney problems [65]. These proteins are thought to be useful markers for predicting how kidney disease and heart problems might progress. Also, lower klotho levels seem to be linked to stiff arteries and heart issues in people with kidney disease [66]. Klotho is generally expressed in the brain, parathyroid gland, and kidney. Klotho is found in the proximal and distal convoluted tubule membrane in the kidney. The extracellular portion of the Klotho membrane protein can be cleaved by a secretase enzyme, generating a soluble fragment known as Kl1, which is released into the bloodstream. Decreased soluble klotho concentrations in the blood and urine could serve as early indicators of CKD, detectable from stage 2 and even earlier in urine in stage 1 [67]. Circulating klotho exerts a cardioprotective effect by inhibiting transient receptor potential canonical type 6 (TRPC6) channels in the heart [68]. This action is mediated through its antagonistic role in the Wnt/β-catenin signaling pathway. TRPC6 is a non-selective cationic channel that exhibits a greater permeability for calcium ions. It prevents hypertrophy by inhibiting the expression of hypertrophic genes, such as B-myosin heavy chain and MHCII, in the heart via calcineurin and NFAT (nuclear factor of activated T cells), as shown in Figure 5.

CKD mice lacking klotho exhibited more severe cardiac hypertrophy after controlling for serum phosphate and FGF23 levels. This suggests that klotho deficiency is a primary contributor to cardiac hypertrophy in CKD, independent of FGF23 and phosphate. In addition to cardiac hypertrophy, klotho deficiency in CKD is implicated in developing cardiac fibrosis [69]. Research has shown a link between LVH and obstructive sleep apnea (OSA), which is associated with upper airway obstruction during sleep. Studies have demonstrated that OSA is independently associated with the development and progression of LVH, likely due to intermittent hypoxia and increased sympathetic activity [70].

### 3.4. Valvular Heart Diseases

Cardiovascular disease is one of the significant causes of death in patients undergoing long-term dialysis. The 1987 study reveals an association between mitral valve abnormalities and CKD [71]. Recent findings (2021–2022) align with the conclusions of a 1987 investigation [72]. Valvular heart disease (VHD) is now an emerging problem for dialysis patients, and ESRD itself increases the risk of developing VHD. Shear stress also hastens the progress of valvular degeneration and increases calcification. Renal replacement therapy is also a significant cause of valvular heart disease, especially mitral valve disease (82%) [73].

Renal replacement therapy affects the heart valves including narrowing (stenosis), leaking (regurgitation), and improper closure (prolapse). These conditions can result from congenital abnormalities, rheumatic fever, infective endocarditis, degenerative changes, or other causes. Valvular heart diseases can lead to symptoms such as dyspnea, chest pain, palpitations, and fatigue, depending on the severity and type of valve involvement [74]. Recent advancements in imaging techniques, particularly echocardiography and cardiac MRI, have improved the diagnosis and management of valvular heart diseases. These modalities provide detailed insights into valve anatomy, function, and blood flow dynamics, guiding treatment choices [75]. Surgical and transcatheter interventions play crucial roles in managing valvular heart diseases, aiming to repair or replace affected valves to restore normal function and alleviate symptoms. Transcatheter mitral valve repair offers less invasive alternatives to traditional surgery, particularly in high-risk or inoperable patients [74]. Despite significant advancements in diagnosis and treatment, VHD remains a significant global health burden, emphasizing the urgent need for research and innovation [75].

### 3.5. Heart Failure

Heart failure (HF) is categorized into chronic HF (CHF) and acute HF (AHF). Acute HF is divided into acutely decompensated chronic HF (ADCHF) and de novo AHF. ADCHF refers to a worsening of existing CHF, while de novo AHF is the sudden onset of new HF symptoms requiring immediate medical attention [76]. Although both are acute forms of HF, they have distinct underlying mechanisms. Decompensated heart failure (DHF) represents a critical deterioration in heart function, necessitating urgent medical intervention. This condition can manifest in both individuals with pre-existing heart failure and those without prior symptoms. The drugs used to treat DHF also affect kidney function and create a situation of cardiorenal syndrome. Heart failure, also known as cardiorenal syndrome type 5, refers to the complex interplay between heart failure and kidney dysfunction in patients undergoing dialysis. CKD and end-stage renal disease (ESRD) are both significant risk factors for the development and exacerbation of heart failure and conversely, heart failure can contribute to worsening kidney function [77]. Heart failure coexists with CKD or with ESRD, as 50% of patients suffering from ESRD undergoing dialysis have the possibility of heart failure. The mechanisms underlying heart failure in dialysis patients are multifactorial and may include volume overload, electrolyte imbalances, anemia, hypertension, and vascular calcification [78].

An interesting aspect of heart failure related to dialysis is the phenomenon known as “cardiorenal connection”. This term describes the bidirectional relationship between the heart and kidneys, where dysfunction in one organ can directly impact the function of the other. For example, impaired cardiac function can lead to decreased renal perfusion and function, while kidney dysfunction can contribute to volume overload and exacerbation of heart failure [50,75]. Renal-replacement therapy and refractory volume overload are suggested as major treatments for ESKD patients to prevent heart failure.

Beta blocker is a common drug used in the treatment of dialysis patients. The study indicates the role of carvedilol as a beta-blocker, which increases left ventricular ejection fraction and reduces left ventricular end-systolic volume and left ventricular end-diastolic volume [79].

### 3.6. SGLT2 Inhibitors and CKD-Associated Heart Failure

Sodium–glucose cotransporter 2 (SGLT2) inhibitors offer a promising new treatment option for individuals with CKD and heart failure (HF) [80]. SGLT2 inhibitors have shown significant efficacy in reducing cardiovascular and renal events in CKD patients. SGLT2 inhibitors can be considered Nobel antidiabetic drugs as SGLT2 inhibitors work by blocking the reabsorption of glucose in the kidneys by blocking the SGLT2 co-transporter located in the apical membrane of tubular cells of the nephron, leading to increased glucose excretion in the urine [81,82,83]. This mechanism not only lowers blood glucose levels but also has several other beneficial effects, such as SGLT2 inhibitors, which can induce a mild diuretic effect by promoting glucose excretion in urine, leading to reduced blood volume and decreased cardiac preload [82]. Lower blood volume can alleviate symptoms of heart failure, such as shortness of breath and fatigue. SGLT2 inhibitors reduce albuminuria [84], a marker of kidney damage, and slow the progression of kidney disease [85]. Xiao-Chun Zeng et al. conducted a study in 2024 to investigate how SGLT2 inhibitors (SGLT2i) reduce albuminuria in patients with type 2 diabetes mellitus (T2DM). They categorized patients into three groups based on their urinary albumin-to-creatinine ratio (UACR): non-albuminuria, microalbuminuria, and macroalbuminuria. After 12 weeks of SGLT2i therapy, the study found a significant reduction in 24 h proteinuria in the macroalbuminuria group. This suggests that SGLT2i may have a beneficial effect on proteinuria in diabetic nephropathy patients. The researchers also explored the potential mechanisms underlying this effect. They found that SGLT2i may reduce oxidative stress in the kidneys but not through the advanced glycation end product (AGE) pathway. Additionally, the study showed that SGLT2i does not activate the renin–angiotensin–aldosterone system (RAAS) [86].

A meta-analysis was conducted to examine the influence of SGLT2 inhibitors on heart failure outcomes and cardiovascular mortality across diverse patient populations and found that SGLT2 inhibitors have been shown to decrease the risk of heart failure events and cardiovascular death in patient populations with heart failure, diabetes mellitus type II, and CKD. These beneficial effects appear consistent across various combinations of these diseases [87].

### 3.7. Arrhythmias

Arrhythmias are a significant concern in patients undergoing renal replacement therapy, particularly hemodialysis. Several underlying conditions and lifestyle factors can increase the likelihood of arrhythmias in this population, including electrolyte imbalances, fluid shifts, sympathetic overactivity, myocardial ischemia, and cardiac fibrosis [88]. One prevalent arrhythmia in dialysis patients is atrial fibrillation (AF). AF is one of the factors that promotes stroke, heart failure, and mortality in dialysis patients [89]. Dysrhythmias in dialysis patients are a common manifestation of mineral and bone disorders, characterized by abnormalities in calcium, phosphate, and parathyroid hormone metabolism. Mineral bone disorder (MBD) is prevalent among individuals with chronic kidney disease (CKD) and is linked to a heightened risk of arrhythmias. Mechanisms contributing to this include myocardial fibrosis, vascular calcification, autonomic dysfunction, and alterations in cardiac ion channels and repolarization [90].

### 3.8. Dilated Cardiomyopathy in Diabetes Patients Associated with CKD

Diabetes significantly increases the risk of cardiovascular complications in patients undergoing dialysis. Individuals with diabetes who develop CKD stage 5 or end-stage renal disease (ESRD) and require hemodialysis face a considerably higher risk of cardiovascular morbidity and mortality compared to those without diabetes [91]. Individuals with diabetes mellitus exhibit an elevated risk of developing atherosclerosis and coronary artery disease due to an accelerated progression of plaque formation within their arterial walls. This heightened risk renders dialysis patients more vulnerable to myocardial infarction and stroke. Furthermore, diabetic nephropathy, a common diabetes complication, significantly contributes to CVD progression among dialysis patients. Diabetic nephropathy and end-stage renal disease synergistically contribute to a complex interplay of metabolic abnormalities and elevate the risk of cardiovascular complications [92].

Interestingly, despite the heightened cardiovascular risk associated with diabetes in dialysis patients, some evidence suggests that diabetes may confer a survival advantage in this population, a phenomenon known as the “reverse epidemiology” or “obesity paradox”. Studies have shown that overweight and obese dialysis patients with diabetes have better survival outcomes compared to their non-diabetic counterparts, although the exact mechanisms underlying this paradoxical association remain unclear and require further investigation [93].

**Figure 6:**
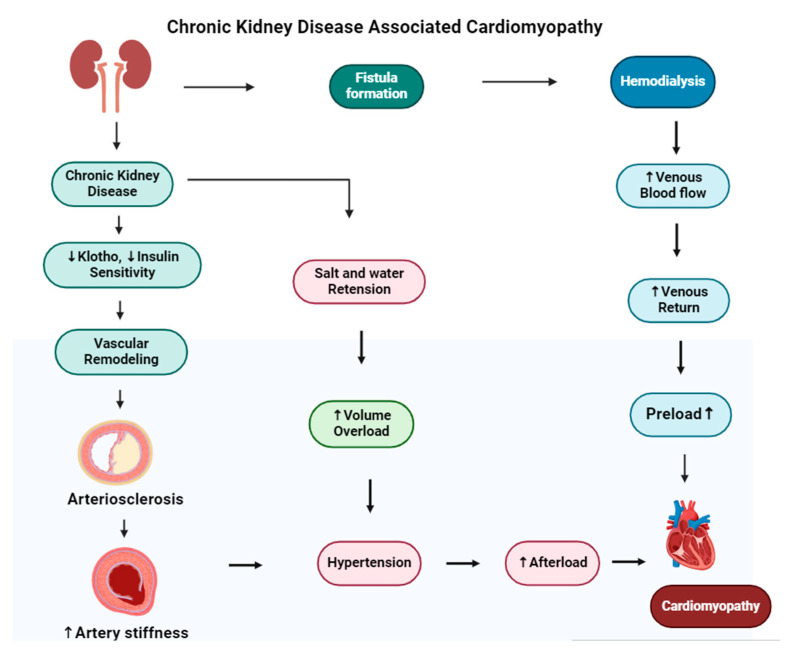
The figure illustrates the development of cardiomyopathy in patients with chronic kidney disease (CKD) undergoing hemodialysis. In the upper pathway, hemodialysis requires vascular access (fistula formation), often created by connecting the radial artery to a vein, which can increase venous return and preload. The lower pathways depict how CKD-related decreases in klotho and insulin sensitivity lead to vascular remodeling and arterial stiffness, contributing to hypertension. Additionally, salt retention and volume overload in CKD can exacerbate hypertension, increasing afterload and ultimately resulting in dilated cardiomyopathy.

A key aspect of the diabetes–dialysis–CVD relationship is “diabetic cardiomyopathy”, characterized by cardiac structural and functional abnormalities independent of coronary artery disease or hypertension. This condition is common among diabetics and, in the dialysis population, can significantly contribute to heart failure and arrhythmias, further increasing the already substantial cardiovascular risk [94]. Chronic kidney disease leads to dilated cardiomyopathy, in which the heart’s muscle stretches and the heart chambers become thin and larger. The mechanism of cardiomyopathy in CKD is shown in Figure 6. Diabetic kidney disease (DKD) is a main health issue and has a risk of adverse health outcomes [95].

### 3.9. Coronary Heart Disease (CHD)

CHD refers to atherosclerotic disease affecting the coronary arteries in individuals undergoing dialysis. Patients with end-stage renal disease (ESRD) are at a higher risk of developing coronary heart disease (CHD). In ESRD, uremia is very common; therefore, uremia along with traditional cardiovascular risk factors have harmful effects on blood vessels, and the accelerated buildup of plaque in the arteries that often occurs with CKD increases the risk of CHD [94].

Dialysis patients with ESRD have a markedly higher prevalence of CHD compared to the general population. This increased risk is evident in the substantially elevated rates of myocardial infarction, angina pectoris, and coronary artery bypass grafting observed in this patient population. The presence of CHD in dialysis patients significantly contributes to cardiovascular morbidity and mortality, representing a major cause of death in this population [96].

An interesting aspect of CHD in dialysis patients is the phenomenon of “dialysis-induced cardiac stunning” or “myocardial stunning”, characterized by transient impairment of myocardial function following hemodialysis sessions. This condition manifests as reversible left ventricular dysfunction, reduced cardiac output, and electrocardiographic changes that mimic myocardial ischemia. Dialysis-induced cardiac stunning is believed to result from acute hemodynamic shifts, electrolyte imbalances, volume fluctuations, and rapid changes in blood pressure during dialysis, highlighting the complex interactions between dialysis treatment and cardiovascular physiology [97].

### 3.10. Obesity as a Risk Factor for Dialysis Patients

Obesity significantly complicates vascular access procedures in dialysis patients. Difficulties arise in creating fistulas or grafts and inserting Tenckhoff catheters, which can be more challenging for CKD patients. Furthermore, obese patients exhibit an increased incidence of catheter malfunctions and peritonitis. In addition, patients need a longer duration for peritoneal dialysis procedures, and dialysis is carried out more frequently to achieve adequate clearance. Therefore, obesity acts as a barrier for dialysis patients for kidney transplantation [98]. A chronic condition characterized by excessive body fat accumulation is intricately linked with an increased risk of cardiovascular disease (CVD). The association between obesity and cardiovascular disease is well-established, although obesity is considered an independent risk factor for chronic kidney disease as excess fatty tissue promotes systemic inflammation and oxidative stress, disrupts lipid metabolism, and alters the secretion of adipokines and cytokines, all of which can adversely affect vascular function and promote atherosclerosis [99].

CKD is characterized by an increase in nitric oxide (NO) and reactive oxygen species (ROS), coupled with a decline in antioxidant defenses and inefficient removal of oxidative byproducts, leading to a state of oxidative stress [100]. During dialysis, antioxidants are lost, and white blood cells are activated, increasing ROS production. This buildup of NO and ROS triggers inflammation, damaging DNA and other biomolecules [98]. Oxidative stress is a major risk factor for cardiovascular disease and increases CKD, particularly in ESRD patients. Oxidative stress is significantly increased in individuals undergoing hemodialysis relative to peritoneal dialysis [101]. Factors such as the dialysis method, membrane biocompatibility, dialysate composition, dialysis duration, and vascular access type all worsen oxidative stress [102]. When the body’s antioxidant defenses are overwhelmed by harmful molecules, this can lead to significant cardiovascular issues in dialysis patients. Furthermore, obesity is strongly associated with the development of left ventricular hypertrophy (LVH), which is associated with diastolic dysfunction, impaired contractility, and increased risk of heart failure [103]. Therefore, it is essential to reduce obesity by exercising, practicing yoga, and consuming a healthy diet to reduce the obesity-induced cardiovascular risk in CKD patients [104] and the latest research indicates that seaweed can be used as an alternative therapy to reduce obesity [105].

### 3.11. Uremic Toxins

Uremic toxins are associated with significant cardiovascular problems in people with ESRD. These toxins negatively affect various cells in the cardiovascular system, including WBC, blood vessel endothelial cells, and thrombocytes [106]. Uremic toxins are classified based on their size (small, middle, and large molecules) and their binding properties (protein-bound and free). Small molecules are readily filtered by healthy kidneys but can accumulate in chronic kidney disease (CKD). Middle molecules, such as β2-microglobulin, are larger molecules that are not efficiently removed by standard dialysis and often require high-flux hemodialysis. Large molecules, primarily proteins, are typically not filtered by the kidneys. Protein-bound toxins, attached to proteins in the blood, are difficult to remove by dialysis. In contrast, free toxins circulate freely and are more readily removed by dialysis [107].

In the presence of uremic toxins, WBCs become less responsive, leading to chronic inflammation, malnutrition, and atherosclerosis. Additionally, uremic states activate macrophages, leading to vascular damage and calcification through oxidative stress and inflammatory pathways [108,109]. Endothelial glycocalyx disruption, increased microvascular permeability, and altered coagulation factors are also consequences of uremic toxin exposure [110]. Uremic toxins further dysregulate vascular function, increase smooth muscle cell proliferation, and promote thrombosis, contributing to cardiovascular complications [111]. Specific toxins like p-Cresol and Indoxyl sulfate accumulate in CKD patients, exacerbating oxidative stress, inflammation, and thrombotic risk, further worsening cardiovascular health [20].

### 3.12. Volume Overload

Excess fluid accumulation constitutes a significant complication of dialysis therapy, directly associated with an elevated risk of cardiovascular events and mortality. Research findings consistently demonstrate that elevated fluid volume serves as an independent prognostic factor for both cardiovascular and all-cause mortality across both peritoneal and hemodialysis patient populations [112]. Furthermore, a large-scale multinational study of hemodialysis patients underscored the critical importance of meticulous fluid management. The study demonstrated a significant increase in mortality risk associated with excess fluid volume, particularly when concurrent with hypotension. Effective volume overload management necessitates maintaining the ideal body weight of dialysis patients through strict adherence to sodium and fluid intake restrictions, coupled with meticulous fluid removal during dialysis sessions [113]. To manage volume overload, it is important to maintain the ideal weight of dialysis patients through sodium and fluid intake restriction and careful fluid removal during dialysis sessions. Interestingly, studies suggest that ultrafiltration rates during dialysis should be carefully monitored and generally not exceed a certain threshold to avoid adverse outcomes, with higher rates associated with increased mortality [114,115]. This underscores the importance of personalized fluid management strategies in dialysis care.

### 3.13. Acid–Base Balance

In ESRD, metabolic acidosis, characterized by low bicarbonate levels, is a frequent complication arising from the decline in renal function. This acid–base imbalance has been associated with systemic inflammation, nutritional deficiencies, and increased mortality risk. Research findings demonstrate a significant association between low bicarbonate levels and increased mortality risk, which is particularly pronounced in patients undergoing peritoneal dialysis. A study involving hemodialysis patients revealed a link between low bicarbonate levels and cardiovascular complications, although no independent association with coronary artery disease or impaired cardiac contractility was observed. Importantly, strategies aimed at correcting metabolic acidosis in ESRD patients, such as dietary modifications and pharmacologic interventions to restore acid–base balance, may offer the potential to improve clinical outcomes and enhance quality of life [116,117].

### 3.14. Dyslipidemia

Dyslipidemia, characterized by an abnormal lipid profile, constitutes a significant cardiovascular risk factor in individuals with CKD, particularly those undergoing dialysis. While dyslipidemia may manifest more prominently in advanced CKD stages (4 and 5), earlier stages can also exhibit atypical lipid profiles, often characterized by elevated triglycerides and reduced high-density lipoprotein (HDL) cholesterol levels, despite seemingly normal or even low total cholesterol levels [118].

Early studies in dialysis patients suggested a potential association between lower cholesterol levels and improved survival outcomes. However, upon further analysis that accounted for confounding factors such as inflammation and malnutrition, the observed relationship between cholesterol and survival in this population mirrored that observed in the general population, suggesting the influence of other contributing factors. Importantly, CKD significantly alters the atherogenic effects of cholesterol, leading to a more pronounced development of atherosclerosis, characterized by severe arterial blockages and heightened inflammatory responses. Studies have demonstrated that the efficacy of cholesterol-lowering medications may be diminished in patients with CKD due to altered physiological processes. Furthermore, CKD can lead to the accumulation of atherogenic lipids, a phenomenon that is more prevalent in patients undergoing peritoneal dialysis. Importantly, current clinical guidelines now recognize CKD as a significant independent risk factor for cardiovascular disease [119,120].

### 3.15. Sclerostin as a Risk Factor for CKD and Cardiovascular Disease

Many factors are responsible for CVD in CKD and ESRD patients, and one novel factor that has been identified is the sclerostin protein. Sclerostin protein is produced by osteocyte cells in the bone and encoded by the SOST gene [121]. The expression of this gene in the heart, kidneys, lungs, and other tissues suggests its involvement in vascular function [122]. Sclerostin inhibits the Wnt pathway in osteoblasts, which is responsible for bone formation. Therefore, sclerostin inhibits bone formation. Although the cardiovascular risk association with sclerostin has not been verified yet in CKD patients, sclerostin levels were found to be higher in CKD patients, which indicates the role of sclerotin in kidney disease. Elevated serum sclerostin and increased sclerostin mRNA expression were observed in patients with aortic valve calcification, which suggests sclerostin plays a major role in aortic valve calcification [123]. Sclerostin was identified as an independent risk factor for heart valve calcification in individuals with CKD and end-stage kidney disease. In a study of 106 hemodialysis patients, cardiovascular risk was associated with higher sclerostin levels (>84 pmol/L) during a five-year follow-up [124].

### 3.16. Hemodialysis and Cardiovascular Risk

In the United States, over 0.4 million patients undergo maintenance hemodialysis treatment. Repeated minor heart damage during hemodialysis treatments may contribute to irreversible heart problems, leading to heart failure and death. The cardiovascular and renal systems interact with each other to maintain stable blood flow, blood volume, and blood vessel tone. When one of these organs malfunctions, both systems can deteriorate over time, known as the cardiorenal syndrome. Hemodialysis patients can develop reno-cardiac syndrome.

#### 3.16.1. Diffuse Myocardial Interstitial Fibrosis

Uremic patients who undergo hemodialysis are affected by specific cardiac fibrosis called diffuse fibrosis and are affected by specific cardiac fibrosis called diffuse myocardial interstitial fibrosis [125]. The myocardial interstitium is the cardiac tissue space where stromal cells and the extracellular matrix (ECM) are located. The ECM includes collagens, elastins, glycoproteins, proteoglycans, hyaluronan, and various signaling molecules. Myocardial fibrosis results from an excessive buildup of collagen fibers, predominantly type I, within the interstitium and around the intramyocardial arteries and arterioles. This imbalance is caused by the overproduction of collagen type I by cardiac fibroblasts and myofibroblasts and the decreased collagen breakdown by extracellular matrix metalloproteinases [126]. Cardiac fibrosis often serves as a reparative mechanism, replacing dead cardiomyocytes with collagen-based scar tissue. In myocardial infarction (MI), the sudden death of heart muscle cells activates reparative myofibroblasts, resulting in scar formation. Although the scar cannot contract, it is essential for maintaining the structural integrity of the heart chamber [127].

#### 3.16.2. Macrovascular Changes

Uremic patients often experience two types of macrovascular changes: atherosclerosis and arteriosclerosis. Atherosclerosis is a disease of the endothelial layer of arteries in which plaque formation, narrowing, and blockage of the vessels lead to impaired blood flow. Patients with ESRD typically have thicker arterial walls and significantly calcified plaques, which leads to chronic myocardial ischemia, followed by chronic myocardial ischemia and myocardial fibrosis.

Arteriosclerosis, a common feature of arterial remodeling in end-stage renal disease (ESRD), is widespread calcification, dilation, and thickening of the middle layer of the aorta and its major branches. This leads to increased arterial stiffness [128]. Disruptions in calcium–phosphorus balance, resulting in low calcium, high phosphate, and secondary hyperparathyroidism, as well as uremic toxins, can accelerate arterial calcification and the conversion of smooth muscle cells into osteocytes [129].

## 4. Conclusions

The kidney in the human body performs various types of functions, including regulating the water and mineral balance. In addition to their primary role in filtering blood, the kidneys function as endocrine organs. They produce erythropoietin, a hormone that stimulates the production of red blood cells, and calcitriol, a hormone that plays a crucial role in bone metabolism [130]. Renin forms a renin–angiotensin system, which maintains the balance of sodium and potassium in the cells and tissue. The antidiuretic hormone secreted from distal convoluted tubules helps water absorption by forming aquaporin channels. Overall, kidneys not only remove waste products but also maintain homeostasis, whereas kidney malfunction produces various complications associated with CVD, such as coronary heart disease, left ventricular hypertrophy, dilated cardiomyopathy, arrhythmia, atherosclerosis, and ultimately, heart failure. Millions of adults in the United States are affected by heart failure, which was responsible for over 370,000 deaths in 2018. The American Heart Association projects that nearly eight million Americans will be living with heart failure by 2030, with one in five individuals aged 40 and older expected to develop the condition [2]. Around one million individuals in the UK are living with symptomatic heart failure. Congestion, a primary factor in the deterioration of renal function in decompensated heart failure, necessitates aggressive decongestion strategies for optimal management.

Therefore, it is mandatory to keep our kidneys healthy to avoid complicated procedures like hemodialysis and peritoneal dialysis, which are used as alternatives to kidneys.

Dialysis is an imperfect substitute for kidney function, as it cannot fully replace the kidney’s role in producing essential hormones. While dialysis effectively removes waste products and excess fluid from the blood, it cannot restore the endocrine functions that the kidneys normally perform. Dialysis procedures rely on highly purified water to ensure optimal treatment. In developed countries, people with CKD also undergo peritoneal dialysis, but the problem associated with peritoneal dialysis is infections in the peritoneum, which can cause peritonitis (a life-threatening condition). Sometimes, the blockage of catheters and hernia is also observed during peritoneal dialysis. Peritoneal dialysis increases the risk of malnutrition in patients as the fluid used in this method can reduce the protein levels in the body, leading to lower energy levels. Therefore, patients with peritoneal dialysis have certain restrictions regarding their diet. To avoid complications in CKD and end-stage kidney disease, proper medication and yoga poses are beneficial.

To prevent CVD in CKD patients, another recent strategy in the latest research is the availability of heart ultrasound at the nephrological clinic. Kidney disease patients often have heart problems, which can lead to serious health issues. Clinicians can use a special type of ultrasound called cardiac ultrasound to check the heart’s health. This test is easier for clinicians who are not cardiologists. Nephrologists can quickly assess the heart’s size, pumping strength, and fluid buildup by learning this technique. This information helps them make better decisions about patient care, both in the hospital and the clinic.

In conclusion, cardiovascular mortality constitutes the principal cause of death in patients with CKD. Although novel therapeutic interventions are being developed to mitigate the cardiovascular risk in CKD patients, the paucity of large-scale cardiovascular outcome trials specifically targeting this high-risk population necessitates further investigation. Rigorous clinical trials focused on individuals with advanced CKD are essential to establish evidence-based strategies for reducing cardiovascular risk and improving outcomes.

## Data Availability

No new data were created or analyzed in this study. Data sharing is not applicable to this article.
